# Intrastriatal injection of interleukin-1 beta triggers the formation of neuromyelitis optica-like lesions in NMO-IgG seropositive rats

**DOI:** 10.1186/2051-5960-1-5

**Published:** 2013-05-08

**Authors:** Maja Kitic, Sonja Hochmeister, Isabella Wimmer, Jan Bauer, Tatsuro Misu, Simone Mader, Markus Reindl, Kazuo Fujihara, Hans Lassmann, Monika Bradl

**Affiliations:** 1 Department of Neuroimmunology, Medical University Vienna, Center for Brain Research, Spitalgasse 4, Vienna, A-1090, Austria; 2 Department of Neurology, Medical University Graz, Graz, Austria; 3 Departments of Multiple Sclerosis Therapeutics and Neurology, Tohoku University Graduate School of Medicine, 1-1 Seiryomachi,Aobaku, Sendai, 980-8574, Japan; 4 Clinical Department of Neurology, Innsbruck Medical University, Anich0strasse 35, Innsbruck, A-6020, Austria

**Keywords:** Neuromyelitis optica, Interleukin-1 beta, Aquaporin 4, NMO-IgG, Blood–brain barrier

## Abstract

**Background:**

Neuromyelitis optica (NMO) is a severe, disabling disease of the central nervous system (CNS) characterized by the formation of astrocyte-destructive, neutrophil-dominated inflammatory lesions in the spinal cord and optic nerves. These lesions are initiated by the binding of pathogenic aquaporin 4 (AQP4)-specific autoantibodies to astrocytes and subsequent complement-mediated lysis of these cells. Typically, these lesions form in a setting of CNS inflammation, where the blood–brain barrier is open for the entry of antibodies and complement. However, it remained unclear to which extent pro-inflammatory cytokines and chemokines contribute to the formation of NMO lesions. To specifically address this question, we injected the cytokines interleukin-1 beta, tumor necrosis factor alpha, interleukin-6, interferon gamma and the chemokine CXCL2 into the striatum of NMO-IgG seropositive rats and analyzed the tissue 24 hours later by immunohistochemistry.

**Results:**

All injected cytokines and chemokines led to profound leakage of immunoglobulins into the injected hemisphere, but only interleukin-1 beta induced the formation of perivascular, neutrophil-infiltrated lesions with AQP4 loss and complement-mediated astrocyte destruction distant from the needle tract. Treatment of rat brain endothelial cells with interleukin-1 beta, but not with any other cytokine or chemokine applied at the same concentration and over the same period of time, caused profound upregulation of granulocyte-recruiting and supporting molecules. Injection of interleukin-1 beta caused higher numbers of blood vessels with perivascular, cellular C1q reactivity than any other cytokine tested. Finally, the screening of a large sample of CNS lesions from NMO and multiple sclerosis patients revealed large numbers of interleukin-1 beta-reactive macrophages/activated microglial cells in active NMO lesions but not in MS lesions with comparable lesion activity and location.

**Conclusions:**

Our data strongly suggest that interleukin-1 beta released in NMO lesions and interleukin-1 beta-induced production/accumulation of complement factors (like C1q) facilitate neutrophil entry and BBB breakdown in the vicinity of NMO lesions, and might thus be an important secondary factor for lesion formation, possibly by paving the ground for rapid lesion growth and amplified immune cell recruitment to this site.

## Background

Neuromyelitis optica (NMO) is a severe demyelinating inflammatory disease of the central nervous system (CNS) [1]. Diagnostic hallmark of NMO is the presence of pathogenic autoantibodies against aquaporin 4 (AQP4) [2], a water channel on astrocytes which is particularly enriched at the perivascular and subpial glia limitans. We and others recently showed that these autoantibodies gain access to their target structures in the course of CNS inflammation mediated by the action of CNS antigen-specific T cells [3-5]. In this experimental paradigm, T cells are needed to open the blood–brain barrier (BBB) for the entry of antibody and complement. We hypothesized that also some cytokines and chemokines, which are produced in the course of brain inflammation, might render the BBB permeable for the entry of antibodies and complement. To address this issue, we used Lewis rats as recipients for intrastriatal injections of cytokines and chemokines, and peripherally challenged these animals with pathogenic antibodies against AQP4 or control IgG. We observed that interleukin-1 beta (IL-1β) was able to trigger the formation of lesions with AQP4 loss outside the needle tract, which was associated with breakdown of the BBB and tissue infiltration by neutrophils. Furthermore, we detected pronounced IL-1β expression in active lesions of NMO patients, but not in stage-matched lesions of multiple sclerosis (MS) patients.

## Results

### Cytokine/chemokine-induced leakage of immunoglobulins across the blood–brain barrier

**Figure 1:**
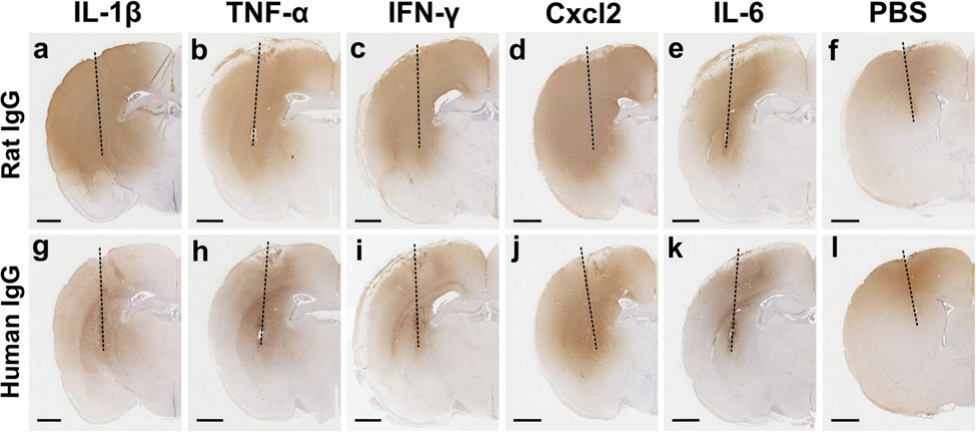
**Blood–brain barrier breakdown induced by the intrastriatal injection of cytokines and chemokines, as indicated by the extravasation of immunoglobulins.** (**a**-**l**) Cerebral hemispheres from juvenile NMO-IgG seropositive Lewis rats stained for the presence of rat immunoglobulins (brown; **a**-**f**) and adjacent sections stained for the presence of human immunoglobulins (brown; **g-l**) 20–24 hrs after intrastriatal injections of 0.3 μl PBS containing 30 ng of IL-1β (**a**,**g**), TNF-α (b,h), IFN-γ (c,i), CXCL2 (**d**,**j**), IL-6 (**e**,**k**) or no further additives (**f**,**l**). The differences of IgG leakage between the different experimental groups was not significant. Dashed line: needle tract. Scale bar: 1 mm.

In first experiments, we injected several different cytokines and chemokines (IL-1β, TNF-α, IFN-γ, CCL7, CX3CL1, CXCL1, CXCL2, and IL-6) into the striatum of juvenile Lewis rats, and analyzed the integrity of the BBB 18–24 hrs later, using rat IgG leakage into the CNS parenchyma as surrogate marker for barrier dysfunction. We found that the injection of IL-1β, TNF-α, IFN-γ, CXCL2, and IL-6 caused profound leakage of rat IgG (data not shown). These findings raised the question whether pathogenic serum antibodies against AQP4 could also enter in sufficient concentrations to initiate damage to astrocytes. To address this point, we next injected these cytokines into the striatum, and at the same time provided NMO-IgG (J0) or human control IgG by intraperitoneal injections as described [4]. 18–24 hrs after injections, the brains of these animals were examined. We found clear evidence for wide-spread leakage of both rat and human IgG (Figure 1), which was not only confined to the injected striatum, but was also seen throughout the entire ipsilateral hemisphere, affecting cortex, corpus callosum, striatum and thalamus (Figure 1). In all cases, the contralateral sides did not show any evidence for IgG leakage (data not shown).

### Entry of inflammatory cells and AQP4 loss at the needle tract

**Figure 2:**
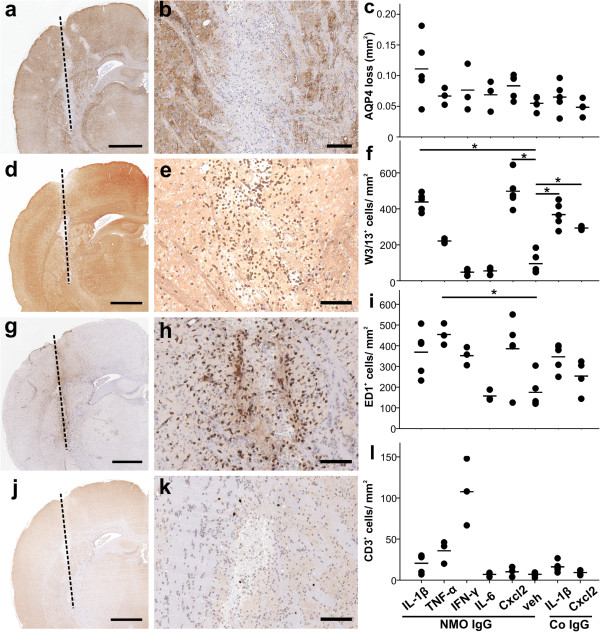
**Pathological findings at the needle tract.** The extent of AQP4 loss (**a-c**) and the infiltration of W3/13^+^ granulocytes (**d-f**), ED1^+^ macrophages/activated microglial cells (**g**-**i**), and CD3^+^ T cells (**j**-**l**) are presented. All histological samples and quantitative data (**c**,**f**,**i**,**l**) shown derive from the striatum of 3-week old Lewis rats injected with 30 ng of IL-1β (histological data), and of IL-1β, TNF-α, IL-6, IFN-γ, CXCL2, or vehicle (PBS, veh) as indicated (quantifications). Animals were seropositive for NMO IgG or control IgG as indicated. Statistically significant differences between individual cytokine treatments are shown (*p<0.05, ANOVA-Dunett T3). Scale bars: 1 mm (**a**,**d**,**g**,**j**) and 100 μm (**b**,**e**,**h**,**k**).

We then studied the effects of this treatment on AQP4 reactivity within the tissue. Only the area immediately adjacent to the needle tract revealed AQP4 loss, but there were no statistically significant differences in the extent of AQP4 loss between the different treatments indicating that these pathological changes at the needle tract were rather caused by the wounding itself than by the action of NMO-IgG at this site (Figure 2). Since all cytokines/chemokines were injected at the same concentration, we next made sure that this concentration was high enough to show biological activity, i.e. to recruit immune cells to the area around needle tract. We found that injection of IL-1β and CXCL2 was associated with a significant recruitment of neutrophils, injection of TNF-α with significant recruitment/activation of macrophages and microglia, and injection of IFN-γ with slight increase in the number of CD3+ T cells, although this did not reach significance (Figure 2). Hence, the cytokine/chemokine concentrations were sufficiently high to reveal effects. Moreover, the effects of IL-1β and CXCL2 were remarkably similar in NMO-IgG seropositive and control IgG positive animals, indicating that, for changes observed at the needle tract, the action of the cytokines was more important than the presence of NMO-IgG.

### Formation of additional, perivascular lesions

Although all the animals described above had comparable AQP4-specific antibody titres in their serum (data not shown), they displayed remarkable differences in the formation of additional lesions:

After intrastriatal injection of CXCL2 and supplementation of the immune system with NMO-IgG, 2/9 animals displayed small perivascular lesions with AQP4 loss and neutrophil infiltration. It remained unclear whether they had formed *de novo*, or whether they represented a continuum with the actual site of injury, since they were found in immediate proximity to the needle tract.

**Figure 3:**
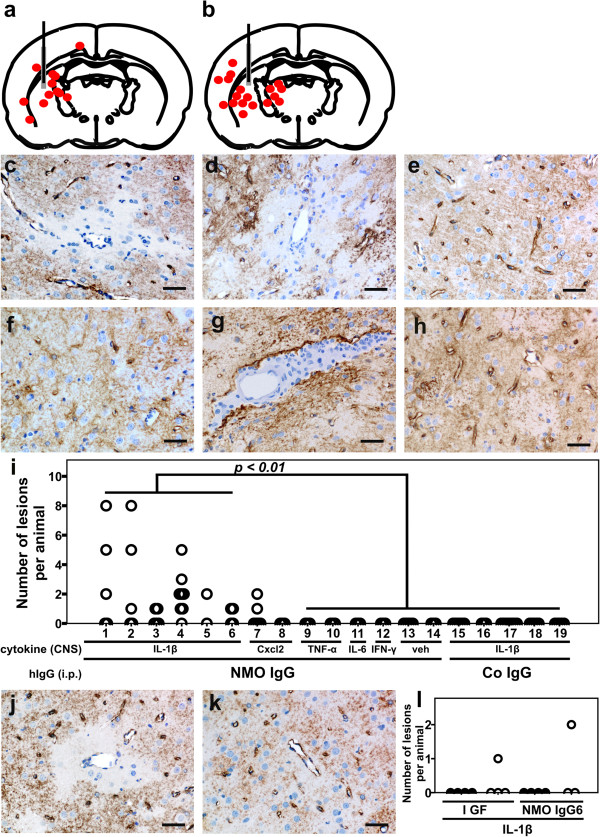
**Location and frequency of lesions with AQP4 loss and neutrophil infiltrates outside the needle tract, after intrastriatal injection of IL-1β and intraperitoneal application of NMO-IgG.** The location (red dots in the schemes of [6]; **a**,**b**) and histology (**c**,**d**) of perivascular lesions with AQP4 loss and neutrophil infiltration in adult animals seropositive for the NMO-IgGs, J0 (**a**,**c**) and I GF (**b**,**d**), that received intrastriatal injection of IL-1β (gray area represents the injection site). Similar lesions were not observed after transfer of IgGs from a NMO-IgG negative NMO patient (**e**), or three NMO-IgG negative MS patients (**f**-**h**). The number of those lesions was significantly higher in IL-1β/NMO-IgG injected animals (n=29) than in the controls (n=47), according to Mann Whitney U test with Bonferroni Holm correction (**i**). The following NMO-IgG or control IgG preparations were used: NMO-IgG J0 (1, 2, 7-14), NMO- IgG I GF (3, 4), NMO-IgG7 (5) and NMO-IgG8 (6). As controls, IgG preparations of two AQP4 antibody negative NMO patients (J3 and J4; 15), three NMO-IgG negative MS patients (J5, J6, J7; 16 and 17), and subcuvia (18, 19) were used. Experiments 1, 3, 7, 9, 11, 12, 13, 15 and 18 were performed using juvenile animals, and experiments 2, 4, 5, 6, 8, 10, 14, 16, 17 and 19 using adult rats. Cytokines were injected as indicated. The AQP4-specific antibodies found in the NMO-IgG preparations are responsible for the formation of lesions, as revealed by absorption studies using two different NMO-IgG preparations (**j**-**l**). Lesions were present (**j**, **l**; white circles) when NMO-IgG had been exposed to emGFP transfected HEK 293 cells, but were absent (**k**, **l**; black circles) when NMO-IgG had been exposed to AQP4-emGFP transfected HEK 293 cells. Bar=25 µm.

However, after intrastriatal injection of IL-1β into NMO-IgG seropositive animals, 3/5 juvenile and 3/5 adult rats showed small perivascular lesions with neutrophilic infiltrates and AQP4 loss, which developed clearly independently from the needle tract-associated area of AQP4 loss (as revealed by careful analysis of consecutive tissue sections). The frequency of these neutrophil-infiltrated lesions with AQP4 loss was highly variable, ranging from 1–8 per lesion-positive rat (Figure 3), which might explain why we also had some animals in each group where we could not detect them at all. In general, these lesions were scattered throughout the entire ipsilateral hemisphere, and were found in the cortex, striatum and thalamus (Figure 3, Additional file 1), but not in the corpus callosum, possibly due to the fact that AQP4 expression is higher in gray matter than in white matter. In all these lesions, the number of CD3^+^ T cells was extremely low (on average 12 cells/mm^2^). Neutrophils (on average 732 cells/mm^2^) and ED1^+^ microglia/macrophages (on average 594 cells/mm^2^) were the dominating cells (Additional file 1). Complement deposition in these lesions was variable (Additional file 2), correlated well with the loss of GFAP^+^ astrocytes or astrocytic foot processes, but was always less pronounced than the complement deposition seen at the needle tract.

There was no evidence for the infiltration of eosinophils, for demyelination or the presence of myelin degradation products in macrophages within these lesions (data not shown), which is in line with the fact that eosinophil recruitment is not a feature of NMO-like lesions in Lewis rats [4], and with the lack of demyelination within a similar time window in previously published NMO models [3,4].

All the data described above have been obtained with NMO-IgG derived from one single patient (J 0). To further substantiate our findings, we repeated these experiments with intrastriatal injection of IL-1β, and intraperitoneal application of IgG from 3 additional anti-AQP4 antibody-positive NMO patients, 2 different anti-AQP4 antibody-negative NMO patients, 3 anti-AQP4 antibody-negative MS patients, and from 2 different AQP4 antibody-positive NMO patients after depletion of AQP4-specific antibodies. Lesions with AQP4 loss and neutrophil infiltration were only observed in the presence of anti-AQP4 antibodies of seropositive NMO patients, but not when AQP4-specific antibodies had been depleted from the NMO-IgG preparations, and also not with any other anti-AQP4 antibody-negative human control IgG (Figure 3).

When IL-1β had been injected into the striatum of human control IgG seropositive animals, neutrophil-infiltrated lesions with AQP4 loss were absent, but small blood vessels with large numbers of intraluminal neutrophils were observed (Additional file 1).

### The effects of IL-1β on endothelial cells of the BBB

**Figure 4:**
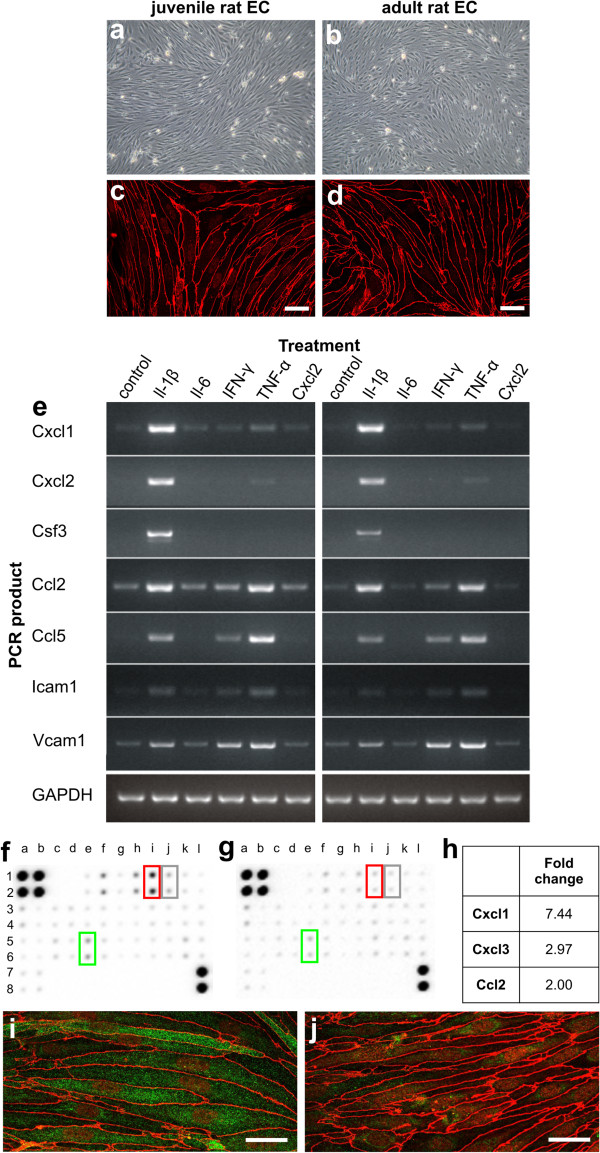
**The effects of cytokines and chemokines on rat brain endothelial cells *in vitro*.** Rat brain endothelial cells (EC) were isolated from the cerebra of 3-week old (**a**,**c**) and adult (**b**,**d**) Lewis rats, and are shown here in phase-contrast microscopy (**a**,**b**) and after staining with an antibody against zonula occludens 1 (ZO-1, red; c, d; scale bar = 25 μm). (**e**) Endothelial cells from 3-week old (left panel) and adult (right panel) Lewis rats were stimulated for 22 hours with vehicle, IL-1β, IL-6, IFN-γ, TNF-α, or CXCL2, and then subjected to PCR analysis of the following genes: Cxcl1, Cxcl2, Csf3, Ccl2, Ccl5, Icam1, Vcam1, and GAPDH. These data are representative of 2 different, independently performed experiments. (**f**,**g**,**h**) The lysates of rat brain endothelial cells treated for 12 hours with IL-1β (**f**) or vehicle (g) were analyzed with antibody arrays and revealed changes in protein expression of CXCL1 (red rectangle), CXCL3 (gray rectangle) and CCL2 (green rectangle). The fold-change in signal intensity of these different proteins was then calculated using the Image J software of the National Institute of Health. (**i**,**j**) Rat brain endothelial cells cultured for 24 hours in the presence of IL-1β (**i**) reveal a marked increase in ICAM1 protein expression (green), which was not seen after 24 hours of culture in the presence of vehicle (**j**). All cells were counterstained with antibodies against ZO-1 (red). Bar = 25 μm. Data are representative of 2 different experiments.

Cytokines/chemokines have only short half-life times [7] and are rapidly redistributed from the parenchyma to the vasculature [8]. Yet, only IL-1β was able to trigger *de novo* formation of perivascular lesions with neutrophilic infiltration and AQP4 loss distant from the needle tract, which indicated that the BBB became permeable for NMO-IgG and complement. To learn more about the mechanisms involved in this process, we cultured rat brain microvascular endothelial cells and confronted these cells for 22 hours with IL-1β, or with other cytokines/chemokines or vehicle as control. We observed that IL-1β was much more efficient in inducing a strong upregulation of mRNA for the granulocyte-recruiting chemokines Cxcl1 and Cxcl2, and for granulocyte colony stimulating factor Csf-3 than any other molecule tested. In addition, IL-1β also triggered enhanced expression of transcripts encoding the monocyte/macrophage-recruiting chemokines Ccl2 and Ccl5, and the adhesion molecules ICAM-1 and VCAM-1 (Figure 4, Table 1). All these effects were identical in endothelial cells derived from juvenile and adult Lewis rats (Figure 4). The increased production of Cxcl1 and Ccl2 by IL-1β could be further confirmed at protein level, using endothelial cell lysates prepared 12 hours after IL-1β treatment (Figure 4), and the expression of ICAM-1 was confirmed by immunocytochemistry on IL-1β-treated endothelial cells (Figure 4).

**Table 1:** Effects of the cytokines on gene expression by rat brain endothelial cells *in vitro* and on tissue pathology *in vivo*

	**Molecules induced in brain endothelial cells *in vitro***					**Effects in the injected brain hemisphere *in vivo***			
**Cytokine**						Perivascular granulocytic	hIgG	perivascular	Perivascular lesions
**Used**	Cxcl1	Cxcl2	Csf3	Icam1	Vcam1	Infiltrates	Leakage	C1q reactivity	With AQP4 loss
**IL-1β**	+++	+++	+++	+	+	++	++	++	++
**TNF-α**	+	+	-	++	++	-	++	+	-
**IFN-γ**	-	-	-	+	+	-	++	-	-
**Cxcl2**	-	-	-	-	-	+	++	-	+ §
**IL-6**	-	-	-	-	-	-	+	-	-
**control**	-	-	-	-	-	-	+	-	-

Semiquantitative analysis of gene expression studies in rat brain endothelial cells isolated from juvenile and adult Lewis rats, and effects in the ipsilateral brain hemisphere of 3 week-old NMO-IgG seropositive Lewis rats treated with/injected with IL-1β, TNF-α, IFN-γ, Cxcl2, IL-6 or vehicle control (PBS). *In vitro* effects revealed strong (+++), intermediate (++), or weak (+) induction of Cxcl1, Cxcl2, Csf3, Icam1, and Vcam1. *In vivo* effects were evaluated by the occurrence of perivascular granulocytic infiltrates and lesions with AQP4 loss, and by the levels of hIgG and C1q reactivity. Please note that only after treatment with IL-1β, all requirements for the formation of *de novo* perivascular lesions with AQP4 loss and granulocyte infiltration are fulfilled.

(§ represents 3 small lesions found in the ipsilateral hemisphere of 6 animals. These lesions were in close proximity to the needle tract area).

### The effects of IL-1β on astrocytes and microglia

We next tested whether IL-1β has also effects on astrocytes and microglial cells *in vitro*. We observed that incubation of astrocytes with IL-1β increased the expression of Cxcl1, Cxcl2, Ccl2, Ccl5 and VCAM-1, while it had little effects on the constitutive expression of ICAM-1 in these cells. In contrast, microglia constitutively expressed Cxcl1, Cxcl2, Ccl2 and ICAM-1 transcripts, possibly due to the baseline activation (and IL-1β production) of these cells under culture conditions. CSF-3 mRNA, however, was not detected in IL-1β treated astrocytes or microglia (Additional file 3).

### The effects of IL-1β on the local availability of complement components

**Figure 5:**
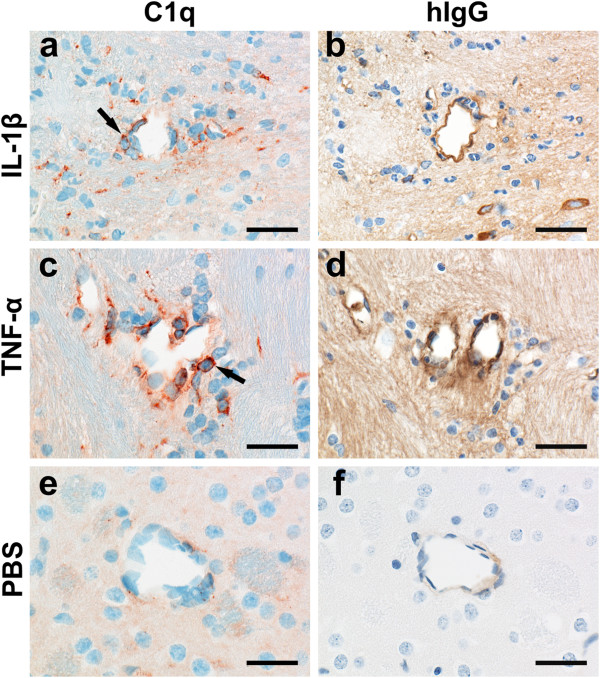
**Perivascular C1q reactivity in the cytokine-injected hemisphere of animals harboring serum NMO-IgG.** Histological analysis (**a**-**f**) of blood vessels with cellular C1q reactivity covering > 50% of the vessel abluminal surface, which were found after the intrastriatal injection of IL-1β (**a**,**b**) and TNF-α (**c**,**d**), but not after injection of vehicle (PBS, e,f), IFN-γ, IL-6 or CXCL2 (not shown). Consecutive sections were stained with antibodies against C1q (red, **a**,**c**,**e**) and human IgG (brown, **b**,**d**,**f**), and counterstained with hematoxylin to reveal nuclei (blue). C1q^+^ glial cells are indicated by arrows. Scale bars: 25 μm.

As described above, lesions with AQP4 loss and neutrophil recruitment showed a variable extent of complement deposition. Therefore, we studied the effects of intrastriatal cytokine injections on perivascular C1q reactivity in NMO-IgG seropositive animals. The largest number of blood vessels with perivascular C1q^+^ cells was observed in animals subjected to IL-1β treatment, followed by animals injected with TNF-α (Figure 5, Table 1). After the injection of IFN-γ, IL-6 or CXCL2, the number of such vessels was similar to PBS-injected control animals (Figure 5, Table 1).

### IL-1β reactivity in active lesions of NMO patients

**Figure 6:**
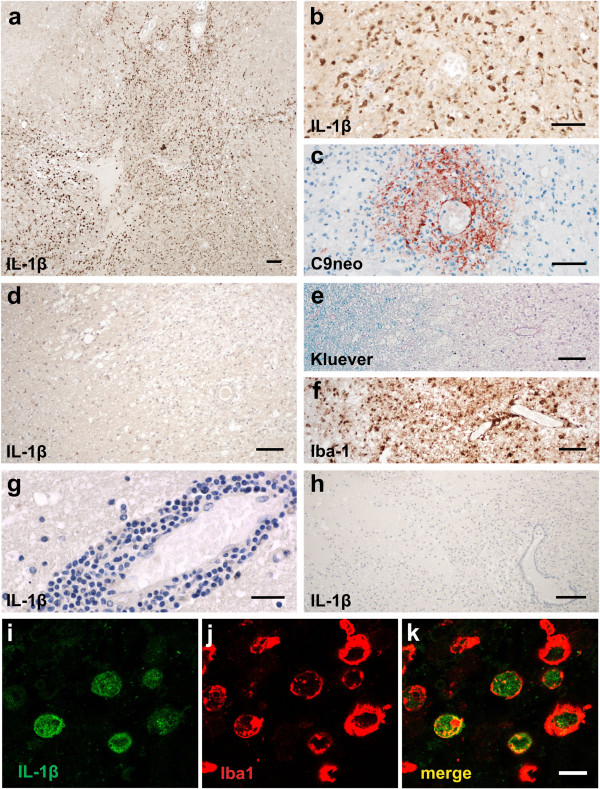
**IL-1β expression in NMO lesions.** IL-1β is expressed in active lesions of NMO patients (**a**). Perivascular lesions with pronounced IL-1β expression (**b**) show the characteristic pattern of complement C9neo deposition (**c**). IL-1β reactivity was absent in active lesions of acute MS cases (**d**,**g**), which were characterized by a high extent of demyelination (e, Kluever staining) and marked microglia activation (f, shown here by Iba1 immunostaining). No signal in NMO lesions was detected upon application of secondary antibody alone (**h**). Confocal microscopy shows macrophages in active NMO lesions stained with IL-1β^+^ (green, i) and Iba1^+^ (red, j). An overlay of these two different stainings is shown in (**k**). Scale bars: 100 μm (**a**,**d**,**e**,**f**,**h**), 50 μm (**b**,**c**), 25 μm (**g**), and 10 μm (**i**-**k**).

We analyzed the expression of IL-1β in human NMO lesions of different stages in comparison to multiple sclerosis lesions and controls. In active NMO lesions, which were characterized by immunoglobulin and complement deposition (Figure 6), massive granulocyte infiltration (Figure 6), AQP4 loss and acute astrocyte and tissue injury (data not shown), we found profound expression of IL-1β in activated macrophages and microglia (Figure 6). This IL-1β expression was restricted to active lesions, while no IL-1β immunoreactivity was detected in more advanced NMO lesions, which lacked complement activation and granulocyte infiltration (Table 2). Furthermore, IL-1β expression was below the level of detection in our immunohistochemical staining of active demyelinating lesions of acute (Figure 6) or chronic MS, or in control brains (Table 2). These differences in IL-1β expression were not a feature of lesion sites (in spinal cords/medullas of NMO patients vs. brains of MS patients), because IL-1β expression was essentially absent in initial/active spinal cord and medullary lesions of MS cases 10 and 11, and in late/active lesions in the spinal cords and optic chiasm of patients with progressive MS (cases 12–17) (Table 2). Moreover, also in EAE, lesions in brain and spinal cord essentially show the same pattern of IL-1β reactivity (data not shown).

**Table 2:** Pathological findings in the CNS of NMO and MS patients and controls

**Case**	**CNS**			**Granulocyte**	**Complement**	**IL-1β**
**number**	**diagnosis**	**CNS area**	**Lesion activity**	**infiltration**	**deposition**	**reactivity**
1	NMO	spinal cord	initial/active	+++	yes	+++
1	NMO	spinal cord	initial/active	+++	yes	+++
2	NMO	medulla oblongata	initial/active	+ (1 area)	yes	+++
3	NMO	spinal cord	initial/active	+++	yes	++
1	NMO	spinal cord	late/active	+	yes	+ (astrocytes)
4	NMO	medulla oblongata	late/active	+	low	++
3	NMO	optic chiasm	late/active	+++	low	++
1	NMO	pons	inactive	-	no	-
1	NMO	mesencephalon	inactive	-	no	-
1	NMO	frontal cortex + WM	inactive	-	no	-
1	NMO	frontal cortex + WM	inactive	-	no	-
5	acute MS	occipital cortex	initial/active	-	no	-
6	acute MS	cerebellum + WM	initial/active	-	no	-
7	acute MS	cortex + WM	initial/active	-	no	-
8	acute MS	parietal cortex + WM	initial/active	+	no	+
9	acute MS	parietal cortex + WM	initial/active	-	no	-
10	acute MS	medulla oblongata	initial/active	-	no	+/− (single cells)
10	acute MS	spinal cord	initial/active	-	no	+/− (single cells)
11	RRMS	spinal cord	initial/active	-	no	-
12	PMS	spinal cord	active	-	no	-
13	PMS	optic chiasm	late/active	-	no	-
14	PMS	spinal cord	late/active	-	no	-
15	PMS	spinal cord	late/active	-	no	+/− (microglia)
16	PPMS	insular cortex + WM	initial/active	-	no	-
17	SPMS	periventricular WM + basal ganglia	chronic	-	no	-
18	HSE	hippocampus	-	-	no	+++
19	No CNS pathology	cortex + WM	-	-	no	-
20	No CNS pathology	temporal cortex + WM	-	-	no	-

Semiquantative analysis revealing different extents (−, +, +++) of granulocyte infiltration, complement deposition and IL-1β reactivity in inflammatory lesions of patients with neuromyelitis optica (NMO), acute multiple sclerosis (MS), primary progressive multiple sclerosis (PPMS), secondary progressive MS (SPMS), Herpes simplex encephalitis (HSE) and control cases without any CNS pathology. WM = white matter.

## Discussion

It is well established that in both NMO patients and animals with NMO/EAE, binding of pathogenic AQP4-specific antibodies to astrocytes paves the way for complement fixation on astrocytes and subsequent destruction of these cells. Unfortunately, the actual mode of entry of these antibodies into the CNS is much less clear. In the healthy CNS, an intact blood–brain barrier seems to efficiently prevent the entry of anti-AQP4 antibodies into the parenchyma. This assumption derives from the observation that NMO patients can be anti-AQP4 antibody positive for many years prior to the onset of the disease [9], and that peripherally administered pathogenic AQP4-specific antibodies do not spontaneously enter the CNS parenchyma of experimental animals [4,10]. However, in the inflamed CNS of rats with NMO/EAE, such NMO patient-derived antibodies gain access to the CNS, find their cellular targets, fix and activate the classical complement cascade [3,4] and trigger the formation of granulocyte-rich, astrocyte-destructive lesions [3-5,11] similar to what is seen just after the direct injection of NMO patient-derived AQP4 specific antibodies and human complement into the mouse brain [12]. In NMO/EAE, the stage for lesion formation is set outside the CNS, by activated CNS antigen-specific T cells, which migrate into the CNS and open the BBB for the entry of cellular and humoral immune mediators at the onset of CNS inflammation. Are there also conditions where the stage for lesion formation is set inside the CNS? We reasoned that this might be indeed the case, and that the local production of IL-1β which is involved in a broad spectrum of CNS diseases [13], might also contribute to the initiation and/or propagation of lesions within the CNS. To specifically address this point, we injected IL-1β and other cytokines/chemokines with known pro-inflammatory functions like TNF-α [14], IFN-γ [15], IL-6 [7,16], and CXCL2 into the striatum, and studied the consequences of this treatment for the recruitment of AQP4-specific antibodies, complement and neutrophils to the CNS. We observed that, in the presence of NMO-IgG in the serum, intrastriatal injection of IL-1β, TNF-α, IFN-γ, and CXCL2 lead to widespread leakage of human IgG into the CNS parenchyma. And yet, only IL-1β caused the formation of perivascular lesions with granulocytic infiltration and AQP4 loss distant from the injection site. The small size of these lesions (in contrast to the highly extensive human NMO lesions) is probably due to a temporally more restricted opening of the BBB in our model, as was also observed in other animal models of EAE [4], but could also demonstrate that additional mechanisms are needed for the formation of NMO lesions besides the presence of NMO-IgG, i.e. the recruitment of granulocytes, the local availability of complement, and sufficient amounts of activated macrophages. Such conditions, for example, are provided in the context of T cell-mediated CNS inflammation [4].

Breakdown of the BBB in response to intrastriatal cytokine/chemokine injections has already been described before [17,18]. It was also observed as a result of intrastriatal injection of adenoviruses expressing IL-1 [19], and could be prevented by neutrophil depletion prior to intrastriatal IL-1β injection [20]. It has also been reported that high levels of TNF-α cause BBB breakdown [21]. Although TNF-α was also able to induce a profound leakage of IgG into the injected hemisphere, we did not see the *de novo* formation of perivascular lesions. The most likely explanations for this finding is the fact that IL-1β acts faster and in much lower concentrations on enthothelial cells than TNF-α [22], and that IL-1β is much more effective in neutrophil recruitment than TNF-α [23]. Granulocytes play an important role in the formation of NMO lesions, since the severity of NMO lesions is increased in mice made neutrophilic, and reduced in mice made neutropenic [24].

IL-1β activates endothelial cells [9,23], which upon IL-1β stimulation produce a number of key molecules leading to the recruitment, production, mobilization, and enhanced survival of neutrophils. Examples of these molecules are Cxcl1 and Cxcl2, Ccl2 and Ccl5, and Csf3 (see our results above, and [25-28]). With the exception of Csf3, these proteins are also produced by IL-1β activated astrocytes [29,30] and microglia [30,31]. They could diffuse through the parenchyma and accumulate at the BBB [8], and could further support the recruitment of neutrophils once the BBB is open. Moreover, IL-1β, TNF-α, and IFN-γ can increase the permeability of the BBB [32,33], which could lead to the leakage of complement proteins into the CNS, and they can induce C1q transcription in microglia [34] and astrocytes [35]. Increased parenchymal synthesis of C1q precedes blood–brain barrier dysfunction [34], C1q can contribute to the endothelial expression of Vcam1 [36], C1q binding to target cell-bound NMO-IgG can trigger antibody-dependent and/or complement mediated cytotoxicity [37,38], and the local activation of complement can trigger neutrophil recruitment [39]. We observed higher numbers of blood vessels with perivascular C1q reactivity in NMO-IgG seropositive rats after the intrastriatal injection of IL-1β and TNF-α.

IL-1β was the only cytokine able to both upregulate neutrophil recruiting/supporting molecules in brain endothelial cells and complement components in brain parenchymal cells. Locally produced and serum-derived complement components might then become activated by NMO-IgG bound to AQP4, and cause NMO lesions.

### What could all these findings mean for NMO patients?

We observed that patients with NMO have significantly more IL-1β expressing macrophages in early active lesions than MS patients with stage-matched lesions do. Although the presence of IL-1β in active MS lesions had been reported elsewhere [40,41], it was below the detection level in a broad spectrum of MS material available in our laboratory, using well-established immunohistochemical methods. On the other hand, the same immunostaining procedure revealed a high number of strongly IL-1β -positive microglia (considerably different from those reported in [40]) in active lesions of NMO patients (Figure 6), as well as in HSE-affected brain tissue (data not shown). The observed pattern of IL-1β reactivity in human CNS material was also confirmed at mRNA level, by in situ hybridization (data not shown). Moreover, other groups also described elevated levels of IL-1β or IL-1β/TNF-α induced molecules like IL-1ra, IL-6, IL-8, IL-13, G-CSF, and Cxcl10 (IP-10) in the CSF of NMO patients, compared to the CSF of patients with multiple sclerosis or other neurological diseases [7,42].

Enhanced IL-1β expression most likely derives from the inflammatory condition *per se*[14,43], where IL-1β is produced by activated microglia cells/macrophages and most likely – in much lower concentrations – also by neutrophils, the most abundant cell type in acute NMO lesions. Although it was for a long time a matter of debate, it is now firmly established that neutrophils do not only transcribe IL-1β mRNA, but are also able to produce and release this protein, essentially only upon stimulation by IL-1β itself or TNF-α [44].

There might be additional amplification mechanims: NMO patients have elevated expression of two key cytokines in their cerebrospinal fluid: IL-6 [7,16] and IL-17 [45]. Both the production of IL-6 by astrocytes, and the stabilization of IL-6 mRNA are triggered by IL-1β [46,47]. IL-17, in turn, prolongs the half-life of the mRNA for CXCL1 [48], a molecule induced in endothelial cells by the action of IL-1β. In addition, IL-17 synergizes with TNF-α to cause an enhanced endothelial expression of neutrophil recruiting chemokines and adhesion molecules [49].

Finally, IL-1β can induce further expression of IL-1β [50,51], which could provide a powerful amplification mechanism to drive neuroinflammatory changes in the brain [13], and it can induce the expression of AQP4 in astrocytes [52], which could increase the amount of target molecules for pathogenic NMO-IgG.

## Conclusions

All the evidence summarized above strongly suggests that the IL-1β released in NMO lesions and the IL-1β-induced local production/accumulation of complement components might facilitate neutrophil entry and BBB breakdown in the vicinity of NMO lesions, and might thus be an important secondary factor for lesion formation, possibly by paving the ground for rapid lesion growth and amplified immune cell recruitment to this site. By doing this, local IL-1β production might play an important role in the propagation and amplification of tissue injury in NMO.

## Methods

### Human CNS samples

Autopsy CNS tissues of patients and control cases archived at the Center for Brain Research, Medical University of Vienna, Austria were used. They included 4 cases with NMO (cases 1–4), 6 cases with acute MS (cases 5–10), 1 case with relapsing remitting MS (case 11), 1 case with primary progressive MS (case 16), 6 case with secondary progressive MS (case 12–15, 17), 1 case with herpes simplex encephalitis (case 18), and 2 cases without evidence of CNS pathology (cases 19 and 20). Studies on archival autopsy tissue were approved by the Ethics Committee of the Medical University of Vienna (EK No. 535/2004/2012).

### Animals

All Lewis rats used in this study were obtained from Charles River Wiga (Sulzfeld, Germany). They were housed in the Decentral Facilities of the Institute for Biomedical Research (Medical University Vienna) under standardized conditions. The experiments were approved by the Ethics Committee of the Medical University Vienna and performed with the license of the Austrian Ministery for Science and Research.

### Injections into the striatum

3-week old (juvenile) and 7-week old (adult) wildtype Lewis rats were anesthetized with Ketanest S/Rompun and injected into the striatum as described [53], using 0.3 μl solution containing 100 ng/μl of the respective cytokines and chemokines in sterile endotoxin-free PBS. The needle was left in place for additional 10 min before it was removed. Immediately afterwards, some rats were left untreated, while the others additionally received an intraperitoneal injection of patient-derived IgG or control IgG (10 mg/ml; 0.5 ml injected in juvenile animals, 1 ml injected in adult animals). The animals were sacrificed 18–24 hrs later for histological analyses.

### Sources of cytokines

The following cytokines were used: rat recombinant IL-1β, IL-6, interferon gamma (IFN-γ), tumor necrosis factor alpha (TNF-α), chemokine (C-X3-C motif) ligand 1 (Cx3cl1) (all from R&D Systems, Minneapolis, MN, USA), rat recombinant chemokine (C-C motif) ligand 7 (Ccl7) and rat recombinant chemokine (C-X-C motif) ligands 1 and 2 (CXCL1 and CXCL2) (all from PreproTech, Rocky Hill, NJ, USA).

### Sources and characterization of patient-derived immunoglobulin preparations

Unless otherwise indicated, experiments were performed with the anti-AQP4 antibody containing human NMO-IgG derived from patient J 0 [4]. We further used human immunoglobulin preparations from patients I GF [4], J NMO-IgG6, J NMO-IgG7, J NMO-IgG8 (all AQP4 antibody-positive NMO patients), J 3 and J 4 (AQP4 antibody-negative NMO patients, [4]) and J 5, J 6, and J 7 (AQP4 antibody negative MS patients, [4]). The use of the patients´ plasma for this study was approved by the Ethics Committee of Tohoku University School of Medicine (No. 2007–327) and the Ethics Committee of Innsbruck Medical University (No. UN3041, 257/4.8).

### Removal of AQP4-specific antibodies from NMO-IgG preparations

NMO-IgG of 2 NMO patients (I GF and J NMO-IgG6) was depleted of AQP4-specific antibodies as described before [4]. This led to a drop in AQP4-specific antibody titers from 1:2560 (untreated NMO-IgG) to 1:1280 (NMO-IgG absorbed with HEK-EmGFP cells) to 1:320 (NMO-IgG absorbed with HEK-AQP4/EmGFP cells) for I GF, and from 1:1280 (untreated NMO-IgG) to 1:640 (NMO-IgG absorbed with HRK-EmGFP cells) to 1:320 (NMO-IgG absorbed with HEK-AQP4/EmGFP cells) for NMO-IgG6.

### Tissue sampling

The animals were sacrificed by inhalation of an overdose of CO_2_. Blood was drawn for the analysis of antibody titers in the serum. Then, the animals were perfused with 4% paraformaldehyde (PFA), the brains were carefully dissected, post-fixed for 24 hrs in 4% PFA, and paraffin embedded.

### Histological analysis

2–4 μm thick adjacent serial sections were cut on a microtome. All stainings were done as described before [54], using the following primary antibodies: polyclonal goat anti-human IL-1β (1:2000, Santa Cruz Biotechnology, Heidelberg, Germany), polyclonal rabbit anti-rat AQP4 (1:250, Sigma-Aldrich, Vienna, Austria), biotinylated sheep anti-human IgG (1:200, Amersham GE Healthcare, Vienna, Austria), donkey anti-rat IgG (1:1500, Jackson Immunoresearch, West Grove, PA, USA), rabbit anti-rat C9neo (1:2000, [55]), rabbit anti-rat C1q (1:100, kindly provided by Sara Piddlesden), polyclonal rabbit anti-cow glial fibrillary acidic protein (GFAP, cross-reactive with rat; 1:3000; DakoCytomation), monoclonal mouse anti-rat ED1 (1:10000), monoclonal rabbit anti-human CD3 (cross-reactive to rat CD3, 1:2000, Thermo Scientific, Vienna, Austria), monoclonal mouse anti-rat W3/13 (1:50, Harlan Sera-Lab), and rabbit anti-Iba1 (1:3000, Wako Chemicals, Neuss, Germany).

Immunohistochemistry was completed by incubation with corresponding biotinylated secondary antibodies (donkey anti-rabbit, 1:2000, sheep anti-mouse, 1:500, both antibodies from Jackson ImmunoResearch; donkey anti-sheep/goat, 1:200, Amersham GE Healthcare), followed by exposure to avidin-peroxidase complex (1:100 in DB/FCS; Sigma). Enhancement of the CD3 staining was performed using biotinylated tyramine amplification [56]. Labeling was visualized with the AEC system (in case of C9neo or C1q) or with 3,3’ diaminobenzidine-tetra-hydrochloride (DAB, Sigma) containing 0,01% hydrogen peroxide. All sections were counterstained with Meyer’s hematoxylin, dehydrated and mounted in geltol (sections developed with the AEC system) or Eukitt (Sigma; all other sections).

For conventional staining, the sections were dewaxed in xylol for 30 min, rehydrated, and stained with hematoxylin/eosin.

For immunofluorescent stainings, the sections were heated for 1 hr in a commercial food steamer using 10 mM EDTA buffer pH 9.0. The sections were then blocked with DAKO Antibody Diluent (DAKO), and goat polyclonal anti-IL-1β (1:125, Santa Cruz Biotechnology) and rabbit anti-Iba1 (1:1500, Wako Chemicals, Neuss, Germany) were applied in the same solution overnight, at 4°C. This was followed by incubation with biotin-conjugated donkey anti-sheep/goat (1:200, Amersham Biosciences), streptavidin-Cy2 (1:75, Jackson ImmunoResearch) and donkey anti rabbit-Cy3 (1:100, Jackson ImmunoResearch) in DB/FCS.

### Quantitative analysis

All histological measurements and cell counts were made using standardized microscopic fields defined by an ocular morphometric grid. To determine the area of AQP4 loss around the injection site, a final magnification of 200 x was used. The grid was positioned over the widest area of the lesion, perpendicular to the needle tract. For final calculation, the area of AQP4 loss caused by wounding (i.e. the needle tract proper) was subtracted from the total area of AQP4 loss. The number of cells recruited to the parenchyma adjacent to the needle tract was also determined at a final magnification of 200 ×. For quantification of cell numbers in newly formed perivascular lesions, a final magnification of 400 × was used, and cross sections of blood vessels were centered in the grid.

### Endothelial cell cultures

Rat brain endothelial cells of 7-9-week old (adult) or 3-week old (juvenile) Lewis rats were cultured essentially as described [57]. To reach high purity of these cultures, 3 μg puromycin (Sigma)/ml culture medium were added [58] for the first 3 days in culture. Afterwards, the culture medium was replaced by endothelial cell medium (PAA) supplemented with 2 ng/ml recombinant human basic fibroblast growth factor (R&D Systems) and 500 ng/ml hydrocortisone (Sigma).

### Immunocytochemical characterization of endothelial cells

Immunocytochemical analysis were done as described [54], using polyclonal rabbit anti-rat zonula occludens 1 (ZO-1, 1:50, Invitrogen by Life Technologies, Vienna, Austria) and monoclonal mouse anti-rat ICAM-1 (1:200, AbD Serotec, Kidlington, UK) as primary, and donkey anti-rabbit Cy5 (1:200, Jackson Immunoresearch) or biotinylated donkey anti-mouse (1:1500, Jackson ImmunoResearch) as secondary antibodies, the latter followed by incubation with DyLight™ 488-conjugated streptavidin (1:75, Jackson ImmunoResearch).

### Cultures of astrocytes and microglial cells

These cells were isolated from neonatal Lewis rats and propagated/purified essentially as described [54].

### Chemokine/cytokine treatment of cells

5-7-day old primary rat brain endothelial cell cultures were washed once with PBS and transferred to endothelial medium without supplements. Since this medium did not contain serum, serum starvation prior to chemokine/cytokine treatment was not necessary. 10 ng/ml of cytokines/chemokines were added, and the culture was continued for 12 (antibody array), 22 (gene expression analysis), or 24 hrs (immunocytochemistry). For antibody array analyses, 1 μl of protein transport inhibitor solution (BD GolgiPlug™, BD Biosciences) was included in the medium. After cytokine treatment, cells were washed three times with PBS and subjected either to RNA or protein isolation, or to immunocytochemical staining.

Microglia and astrocytes were cultured under serum-free conditions over night, and were then incubated with 10 ng/ml IL-1β for 22 hrs.

### Antibody array analysis

Cells were washed with PBS. Proteins were isolated and analysed using the RayBio® Rat Cytokine Array 2 kit (RayBiotech, Norcross, GA, USA) according to the manufacturer’s instructions.

### RNA isolation and cDNA synthesis

RNA was isolated with the RNeasy kit and QIAshredder (both from Qiagen, Vienna, Austria) according to the instructions of the manufacturer. First strand cDNA was synthesized using M-MLV Reverse Transcriptase (Promega, Mannheim, Germany), as suggested by the manufacturer. Afterwards, the cDNA was used directly for polymerase chain reactions (PCR).

### PCR analysis

The following primer pairs were used: Cxcl1 (forward 5^′^-AAGGGTGTCCCCAAGTAATGG-3^′^; reverse 5^′^-CCTTCTTCCCGCTCAACACC-3^′^), Cxcl2 (forward 5^′^-CACCAACCATCAGGGTACAGG-3^′^; reverse 5^′^-GAGGCACATCAGGTACGATCC-3^′^), CSF-3 (forward 5^′^-TTGCCACCACCATCTGGC-3^′^; reverse 5^′^-ACTGCTGTTTAAATATTAAACAGGG-3^′^), Ccl2 (forward 5^′^-CACTCACCTGCTGCTACTCATTCA-3^′^; reverse 5^′^-GCTTGAGGTGGTTGTGGAAAAG-3^′^), Ccl5 (forward 5^′^-CTGCTGCTTTGCCTACCTCTCC-3^′^; reverse 5^′^-GATAGCATCTATGCCCTCCCAGG-3^′^), Icam1 (forward 5^′^- GGGTTGGAGACTAACTGGATGA-3^′^; reverse 5^′^-GGATCGAGCTCCACTCGCTC-3^′^), Vcam1 (forward 5^′^-GAGACAAAACAGAAGTGGAAT-3^′^; reverse 5^′^-AGCAACGTTGACATAAAGAGT-3^′^) and GAPDH (forward 5^′^-GGCATTGCTCTCAATGACACC-3^′^; reverse 5^′^-TGAGGGTGCAGCGAACTTTAT-3^′^). The FastStart Taq DNA Polymerase kit (Roche Applied Science, Vienna, Austria) was used for amplifications. One reaction consisted of: 5 μl 10x PCR buffer (200 mMTris–HCl, pH 8.4, 500 mM KCl), 1 μl 10 mM dNTP mix, 1 μl forward primer (100 pmol/μl), 1 μl reverse primer (100 pmol/μl), 0.4 μl polymerase (5 U/μl), 1 μl cDNA and 40.6 μl H_2_O. The reaction mixture was subjected to an initial denaturation step (11 min, 95°C), and then to 25, 28, 30 or 35 cycles of denaturation (30 s, 95°C), annealing (30 s; 55°C for Cxcl1, 57°C for Cxcl2 and Csf3, 56°C for Ccl2 and Icam1, 59°C for Ccl5, 49°C for Vcam1, and 53°C for GAPDH) and elongation (30 s, 72°C). The reaction was terminated with final extension for 10 min at 72°C, and PCR products were detected by agarose gel electrophoresis.

### Statistics

The following statistical evaluations were performed using the PASW statistics 18 software system (SPSS Inc., Chicago, USA): One-way ANOVA, followed by Dunett T3 post-hoc test, and Kruskal-Wallis followed by Mann–Whitney U test and Bonferroni-Holm correction.

## Competing interests

The authors declare that they have no competing interests.

## Authors’ contributions

MK performed immunohistological analysis. She established propagated and treated astrocyte, endothelial cell and microglial cultures, analyzed these cells by immunocytochemistry, PCR, and antibody arrays, and drafted the manuscript. SH performed the intrastriatal injections; IW established endothelial cell cultures; JB established IL-1β stainings of human CNS tissue sections; TM selected and purified NMO-IgGs; SM purified NMO-IgGs and determined the antibody titers; MR selected NMO-IgGs and coordinated the NMO-IgG purification and absorption; KF selected NMO-IgG and participated in the coordination of this study; HL and MB conceived this study and helped to draft the manuscript. All authors read and approved the final manuscript.

## Supplementary Material

Additional file 1 **Loss of AQP4 and neutrophil infiltration in perivascular areas distant from the needle tract.** (a-d) The location of lesions with AQP4 loss and neutrophil infiltration in IL-1β injected, NMO-IgG seropositive juvenile (a,c) and adult rats (b,d). The lesions were observed in the cortex, striatum and thalamus (see boxes in a and b), but were not observed in the contralateral hemisphere. (c) Cortical lesion boxed in (a), insert shows neutrophils. (d) thalamic lesion boxed in (b). (e-f) When IL-1β has been administered to the striatum of animals carrying human control antibodies instead of NMO-IgG in their serum, vessels with larger numbers of intraluminal neutrophils were observed, while there were almost no neutrophils in the surrounding parenchyma. Tissue sections were stained for AQP4 (brown), and counterstained with hematoxylin to reveal nuclei (blue). The needle tract is shown by the dashed line. The black arrows point to neutrophils. Bar = 25 μm. Click here for file

Additional file 2 **Differences in C9neo deposition and astrocyte loss between lesions.** Locations of representative ipsilateral lesions (a, b) or corresponding contralateral blood vessel (c), of IL-1β-injected animal, are depicted in schemes. Serial sections of those lesions are shown after stainings for C9neo (red, d, e, f), human IgG (brown, g, h, i), AQP4 (brown, j, k, l), GFAP (brown, m, n, o), W3/13 (brown, p, q, r) and ED1 (brown, s, t, u). The sections were counterstained with hematoxylin to reveal nuclei (blue), and represent lesions ipsilateral close (a,d,g,j,m,p,s) and distant (b,e,h,k,n,q,t) to the needle tract, or, for comparison, blood vessels found at the contralateral side (c,f,i,l,o,r,u,). Note, that lesions closer to the needle tract are characterized by higher levels of complement deposition, by more severe astrocytic damage (i.e. loss of GFAP reactivity), and by the recruitment of more W3/13^+^ cells (granulocytes and T cells) than their more distant counterparts. Bar = 25 μm. Click here for file

Additional file 3 **Effects of IL-1β on microglia and astrocytes *in vitro*.** Microglia and astrocytes were stimulated for 22 hours with IL-1β or vehicle, and then subjected to PCR analysis of the following genes: Cxcl1, Cxcl2, Ccl2, Ccl5, Icam1, Vcam1, and GAPDH. Click here for file
