# Supplementary material for: Intrastriatal injection of interleukin-1 beta triggers the formation of neuromyelitis optica-like lesions in NMO-IgG seropositive rats
Source: Acta Neuropathol Commun. 2013 May 8;1:5. doi: 10.1186/2051-5960-1-5 (PMC3776214; doi:10.1186/2051-5960-1-5)
Supplement: Additional file 1 — Loss of AQP4 and neutrophil infiltration in perivascular areas distant from the needle tract. (a-d) The location of lesions with AQP4 loss and neutrophil infiltration in IL-1β injected, NMO-IgG seropositive juvenile (a,c) and adult rats (b,d). The lesions were observed in the cortex, striatum and thalamus (see boxes in a and b), but were not observed in the contralateral hemisphere. (c) Cortical lesion boxed in (a), insert shows neutrophils. (d) thalamic lesion boxed in (b). (e-f) When IL-1β has been administered to the striatum of animals carrying human control antibodies instead of NMO-IgG in their serum, vessels with larger numbers of intraluminal neutrophils were observed, while there were almost no neutrophils in the surrounding parenchyma. Tissue sections were stained for AQP4 (brown), and counterstained with hematoxylin to reveal nuclei (blue). The needle tract is shown by the dashed line. The black arrows point to neutrophils. Bar = 25 μm. [file 2051-5960-1-5-S1.pdf]

Addional file 1.pdf:

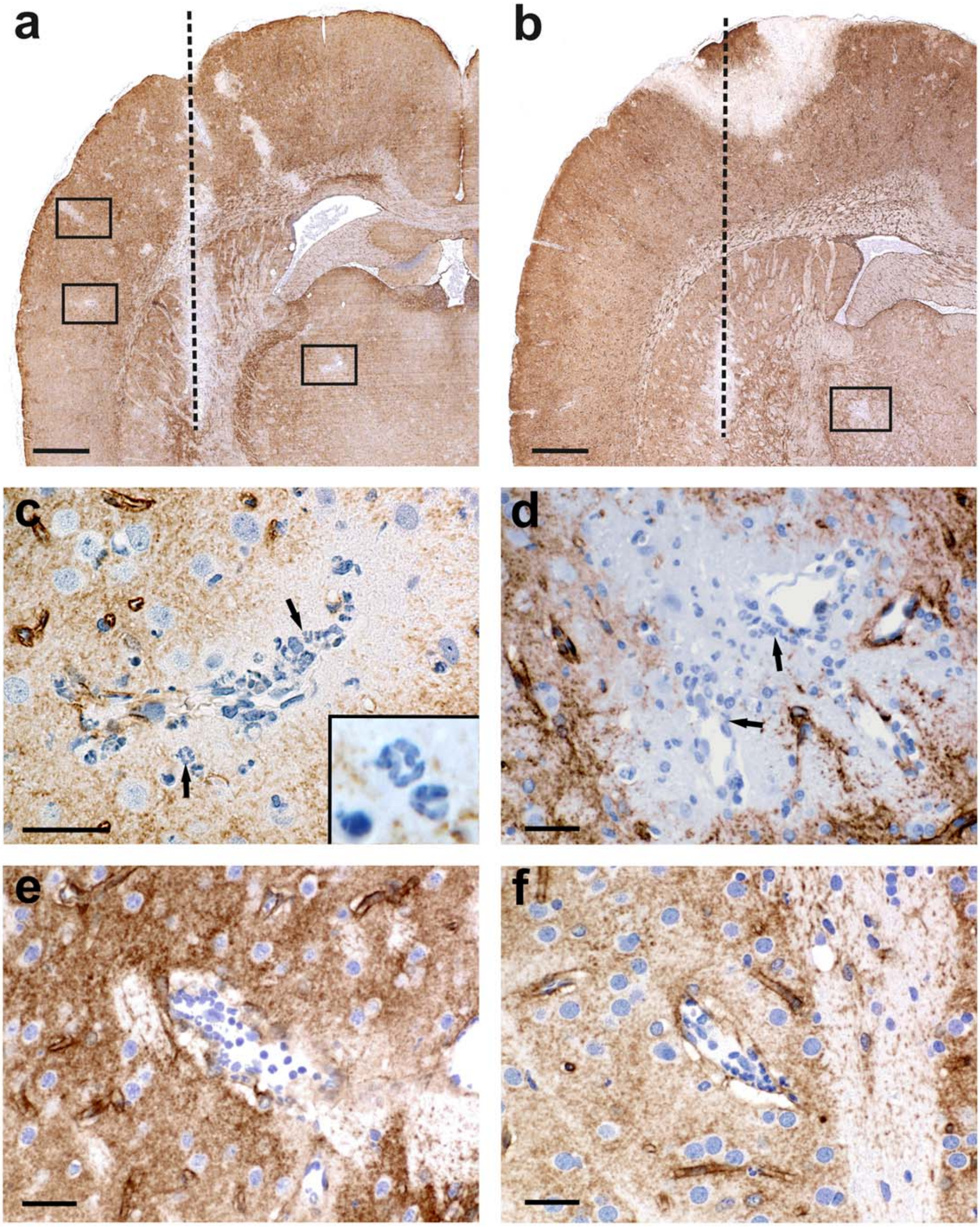

Loss of AQP4 and neutrophil infiltration in perivascular areas distant from the needle tract.

(a-d) The location of lesions with AQP4 loss and neutrophil infiltration in IL-1β injected, NMO-IgG seropositive juvenile (a,c) and adult rats (b,d). The lesions were observed in the cortex, striatum and thalamus (see boxes in a and b), but were not observed in the contralateral hemisphere. (c) Cortical lesion boxed in (a), insert shows neutrophils. (d) thalamic lesion boxed in (b). (e-f) When IL-1β has been administered to the striatum of animals carrying human control antibodies instead of NMO-IgG in their serum, vessels with larger numbers of intraluminal neutrophils were observed, while there were almost no neutrophils in the surrounding parenchyma. Tissue sections were stained for AQP4 (brown), and counterstained with hematoxylin to reveal nuclei (blue). The needle tract is shown by the dashed line. The black arrows point to neutrophils. Bar = 25 µm.
