# Supplementary material for: Intrastriatal injection of interleukin-1 beta triggers the formation of neuromyelitis optica-like lesions in NMO-IgG seropositive rats
Source: Acta Neuropathol Commun. 2013 May 8;1:5. doi: 10.1186/2051-5960-1-5 (PMC3776214; doi:10.1186/2051-5960-1-5)
Supplement: Additional file 2 — Differences in C9neo deposition and astrocyte loss between lesions. Locations of representative ipsilateral lesions (a, b) or corresponding contralateral blood vessel (c), of IL-1β-injected animal, are depicted in schemes. Serial sections of those lesions are shown after stainings for C9neo (red, d, e, f), human IgG (brown, g, h, i), AQP4 (brown, j, k, l), GFAP (brown, m, n, o), W3/13 (brown, p, q, r) and ED1 (brown, s, t, u). The sections were counterstained with hematoxylin to reveal nuclei (blue), and represent lesions ipsilateral close (a,d,g,j,m,p,s) and distant (b,e,h,k,n,q,t) to the needle tract, or, for comparison, blood vessels found at the contralateral side (c,f,i,l,o,r,u,). Note, that lesions closer to the needle tract are characterized by higher levels of complement deposition, by more severe astrocytic damage (i.e. loss of GFAP reactivity), and by the recruitment of more W3/13+ cells (granulocytes and T cells) than their more distant counterparts. Bar = 25 μm. [file 2051-5960-1-5-S2.pdf]

Additonal file 2.pdf:

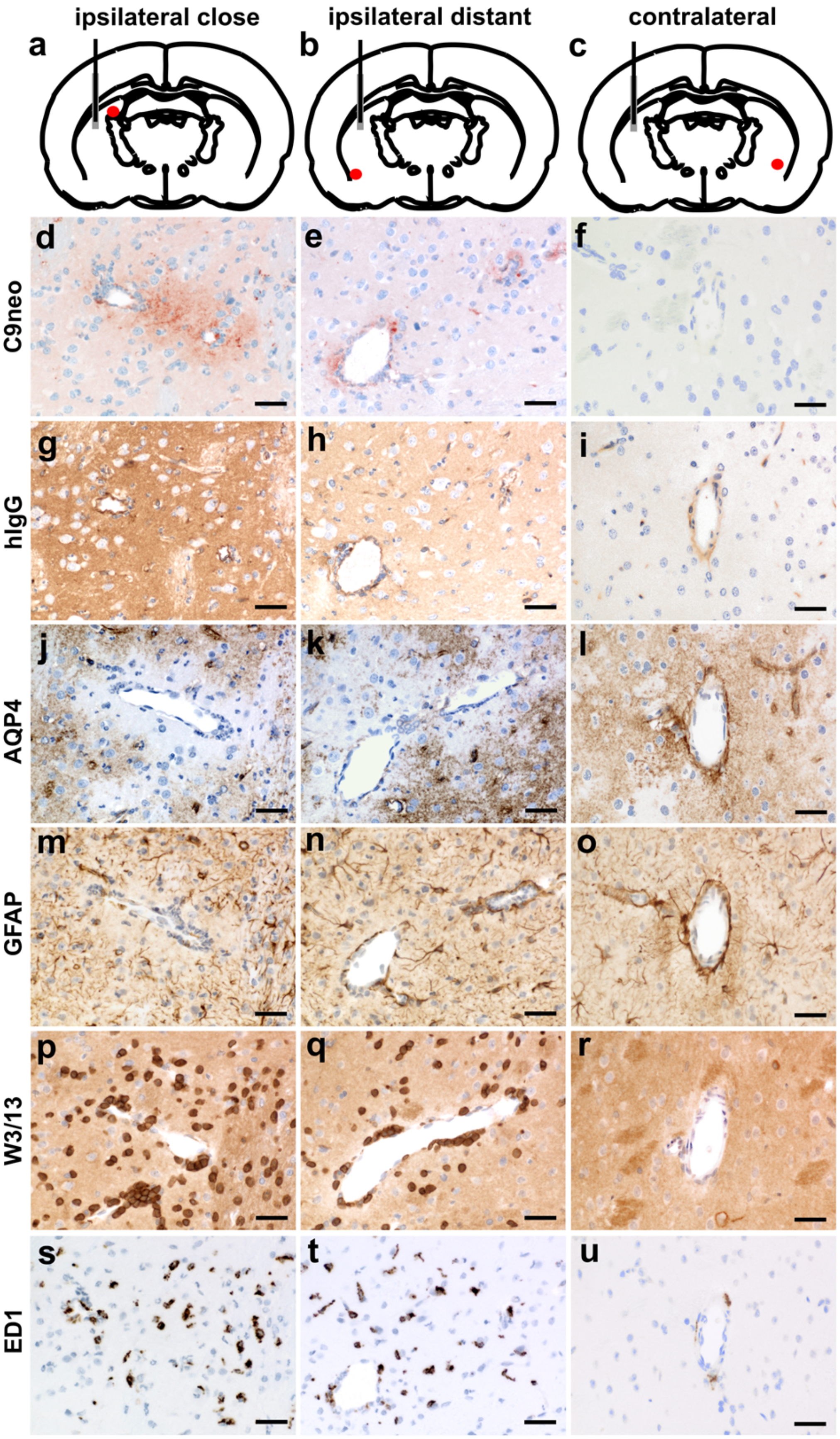

Differences in C9neo deposition and astrocyte loss between lesions.

Locations of representative ipsilateral lesions (a, b) or corresponding contralateral blood vessel (c), of IL-1β-injected animal, are depicted in schemes. Serial sections of those lesions are shown after stainings for C9neo (red, d, e, f), human IgG (brown, g, h, i), AQP4 (brown, j, k, l), GFAP (brown, m, n, o), W3/13 (brown, p, q, r) and ED1 (brown, s, t, u). The sections were counterstained with hematoxylin to reveal nuclei (blue), and represent lesions ipsilateral close (a,d,g,j,m,p,s) and distant (b,e,h,k,n,q,t) to the needle tract, or, for comparison, blood vessels found at the contralateral side (c,f,i,l,o,r,u,). Note, that lesions closer to the needle tract are characterized by higher levels of complement deposition, by more severe astrocytic damage (i.e. loss of GFAP reactivity), and by the recruitment of more $W3/13^{+}$ cells (granulocytes and T cells) than their more distant counterparts. Bar = 25 µm.
