# Supplementary material for: Intrastriatal injection of interleukin-1 beta triggers the formation of neuromyelitis optica-like lesions in NMO-IgG seropositive rats
Source: Acta Neuropathol Commun. 2013 May 8;1:5. doi: 10.1186/2051-5960-1-5 (PMC3776214; doi:10.1186/2051-5960-1-5)
Supplement: Additional file 3 — Effects of IL-1β on microglia and astrocytes in vitro. Microglia and astrocytes were stimulated for 22 hours with IL-1β or vehicle, and then subjected to PCR analysis of the following genes: Cxcl1, Cxcl2, Ccl2, Ccl5, Icam1, Vcam1, and GAPDH. [file 2051-5960-1-5-S3.pdf]

Additional file 3.pdf:

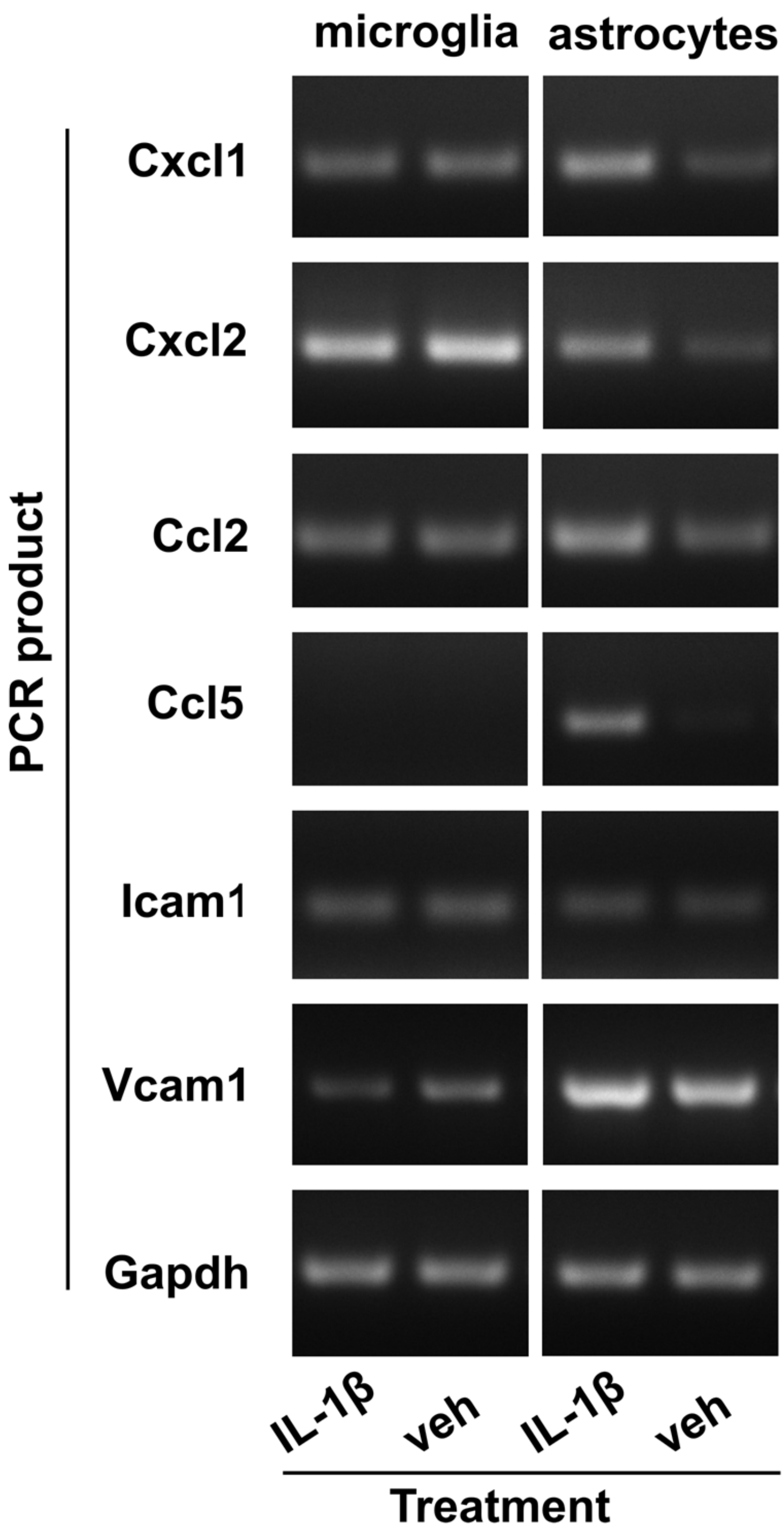

Effects of IL-1β on microglia and astrocytes in vitro

Microglia and astrocytes were stimulated for 22 hours with IL-1β or vehicle, and then subjected to PCR analysis of the following genes: Cxcl1, Cxcl2, Ccl2, Ccl5, Icam1, Vcam1, and GAPDH.
